# Protocol for a systematic review on the experience of informal caregivers for people with a moderate to advanced dementia within a domestic home setting

**DOI:** 10.1186/s13643-020-01525-0

**Published:** 2020-11-26

**Authors:** Charles James, Catherine Walshe, Katherine Froggatt

**Affiliations:** Faculty of Health and Medicine, Lancaster University, Bailrigg, Lancaster, LA1 4YG UK

**Keywords:** Informal caregiver, Dementia, Moderate dementia, Advanced dementia, Moral distress, Internal conflicts

## Abstract

**Background:**

The knowledge about the experience of informal caregivers who provide care to people with moderate to advanced dementia in a domestic home setting is limited. A consequence of long hours of caregiving in addition to dealing with normal challenges of daily living is their experience of a poor quality of life*.* Some of their experiences may be described in terms of a feeling of powerlessness to make changes during care provision. This feeling may also suggest an experience of moral distress. The aim of this systematic review is to synthesise qualitative evidence relating to these experiences.

**Methods:**

This review adopts a narrative synthesis approach. A search will be conducted for studies written in the English language in the bibliographic databases MEDLINE Complete, CINAHL, EMBASE, PsycINFO, Web of Science and Academic Search Complete covering periods from 1984 to present. Included studies will be qualitative or mixed-methods designs. The search terms will be related to dementia and caregivers, and the process will be focused on dementia at the moderate to the advanced stages within the domestic home setting. Reference lists of included papers will also be searched for additional relevant citations. Search terms and strategies will be checked by two independent reviewers. The identification of abstracts and full texts of studies will be done by the author, while the quality and the risk of bias will also be checked by the two independent reviewers.

**Discussion:**

Psychological distress is cited as an experience reported within informal caregiving. For the caregiver, it is associated with a negative impact on general health. To date, no synthesis exists on the specific experience of informal caregiving for people with moderate to advanced dementia within the domestic home setting. This review considers that variation of accounts contributes to how the informal caregivers’ general experience is explored in future research. This may enable gaps in current knowledge to be highlighted within the wider context of caregiving in the domestic home setting.

**Systematic review registration:**

This review is registered with PROSPERO (CRD42020183649).

**Supplementary Information:**

The online version contains supplementary material available at 10.1186/s13643-020-01525-0.

## Background

Dementia is described as a progressive disease that affects cognitive abilities and causes everyday functional capabilities to deteriorate [10, 16]. The main types of this disease include Lewy body dementia, frontotemporal dementia, vascular dementia and mixed dementia [22]. Its most common cause has also been identified as Alzheimer’s disease [4]. As a cure or an effective prevention is still unknown, the disease is regarded as a disease of increasing public health concern [41]. By 2014, around 850,000 people had been affected [24]. Of this population, 1 in 6 people was 80 years of age, and over 40,000 people under the age of 65 were also affected [24]. Regardless of the age, the overall number of people with dementia is also predicted to rise to over 2 million by 2051 unless a cure is found [24, 28].

Several advancements have been made in recognition of the symptoms and diagnosis of dementia, although evidence also indicates that caring for people affected is primarily done by their families [8]. There are approximately 670,000 people who act as family caregivers [14], and it has been suggested that the amount of care given by these caregivers increases in line with the severity of the disease [31]. As a result, the burden of care over the disease trajectory is often also increased [31].

Previous studies have shown that the burden of care is associated with an increasing severity of the decline in dementia, especially between the moderate to the advanced stages [9, 19]. In the moderate stage, the caregiver’s needs are often twice as many as those of the care recipient’s due to the increasing functional decline as the disease progresses [9, 23]. The decline is experienced by the care recipient as an increasing loss of functional capability to self-care in daily living activities such as washing, feeding and mobilising. A simultaneous increase in the management of behavioral and psychological symptoms of the disease is also experienced by the caregiver as a challenge which impacts negatively in terms of burden and psychological distress [18].

The care recipient’s increased inability to self-care also extends into the advanced stages of dementia and through to the end of life [46]. This also leads to a higher level of reliance on the caregiver than in previous stages [30]. Unlike other terminal diseases such as cancer, prediction of life expectancy in dementia is however often difficult [5, 20]. In most cases, up to 10 years after diagnosis is a common assumption [52]. Thus, it is likely that the burden of caring is also experienced by the informal caregivers for most of this extended period due to caregiving responsibility changes across the stages. As such, while it might be preferred to care at home for people in the later stages of dementia, most informal caregivers will opt to employ someone else to provide the necessary care [8].

Entering the advanced stages of dementia also signifies a further progression through the trajectory of the disease. Moreover, as this indicates that a care recipient may be approaching the later stages of the disease where the end of life care is necessary, caregivers also experience an increased level of anxiety and burden [8, 43]. It has been highlighted that this may be due to the likelihood of institutionalisation at this stage, which is also a cause of caregiver’s psychological distress [8, 12, 17]. Indeed, available evidence shows that between 2001 and 2010, a higher proportion of people with dementia die in environments other than their domestic home, such as in hospitals and residential care homes [48].

It has been suggested that the number of people with dementia who require palliative care will grow four folds by 2040 [15]. Evidence also shows, however, that currently, around 850 informal caregivers are providing round-the-clock unpaid care for every 1000 people living with dementia within the domestic home setting [8, 25, 36]. These informal caregivers, often referred to as the ‘invisible second patients’, given the long hours of caregiving, collectively save the UK economy an estimated £132 billion annually in their unpaid capacity [27]. They experience a low quality of life in addition to coping with the usual daily living challenges [8]. As a result, the effect on their well-being indicates that their psychological distress has a negative impact on their physical wellbeing [7, 26, 34, 50].

Emotional distress, depression and an increasing feeling of isolation are some of the experiences that have a direct impact on the health of the informal caregivers [8]. Heart-related physical illnesses and other chronic disorders have also been reported [8]. However, some studies have argued that caring may also be defined in terms of the informal caregiver’s sense of fulfilment in facilitating a good end of life for a member of the family [8, 49]. In this regard, examples of positive experiences have also been given, some of which indicate that continued care provision may be viewed as the personal choice of informal caregivers [3]. It is also evident, however, that the number of informal caregivers caring within the domestic home setting continues to increase regardless of the reported cases of psychological distress [39].

Placing the care recipient in other care settings than the domestic home is however often considered due to the negative experiences of psychological distress experienced by the informal caregiver [7, 8]. It is also argued that when behaviours become unmanageable, institutionalisation may be considered at a stage in the disease trajectory [47]. With this perspective, the willingness of the informal caregiver to continue caregiving within the domestic home setting may be considered in terms of fulfilling a moral obligation. It is also plausible to consider the negative experiences reported as a resultant influence from a psychological coercion borne out of devotion or a feeling of loss of a loved one. Such feeling has been highlighted in terms of a personal tragedy and associated with a disability such as dementia [1, 37, 38]. Alternatively, it may also give evidence of an internal conflict encountered while caring at the end of life [13].

Previous studies have adopted varying methodological approaches that indicate that informal caregivers have both positive and negative experiences of caring for people living with dementia. In order to explain the specific experiences of care provision within the moderate to advanced stages of the disease, a consensus is required to also understand the methodologies currently adopted in this area of interest. It is recognised that while positive accounts can be provided as part of the experience of caring for people with dementia, caring in an unpaid capacity has resulted in a poor quality of life. This review seeks to understand the current knowledge of those experiences unique to informal caregivers within the domestic home setting for people with a moderate to advanced dementia. The gaps that remain in knowledge are also explored.

## Methods

The present protocol has been registered within the PROSPERO database (registration number CRD42020183649) and is being reported in accordance with the reporting guidance provided in the Preferred Reporting Items for Systematic Reviews and Meta-Analyses Protocols (PRISMA-P) statement (see checklist in Additional file 1). This review adopts a narrative synthesis approach. Narrative synthesis approaches systematic reviews by using words and texts to synthesize and explain findings from a combination of disparate study designs [40]. As a systematic approach which favours the presentation of findings from theoretically established literature within an area of interest, it is useful in summarising the current state of knowledge about a review question [40]. In this review, the aim is to identify any existing literature and current knowledge about the experience of informal caregivers who provide care to people with moderate to advanced dementia within a domestic home setting.

In consistency with addressing areas within the scope of interest, exploration of the key review question will be conducted within the contexts of dementia, informal caregivers and domestic home setting. The main findings from the synthesis of identified studies will then be used in exploring these experiences within the moderate to advanced stages of the disease within the domestic home setting. By adopting an iterative approach in the process, the link between the data from the findings and the individual studies identified will be mapped [40]. The criteria for identifying which studies to include will also be guided by the different components within the review question.

### Review aims and question

The aim of this review is to explore informal caregivers’ experiences of home-based caregiving for people with a moderate to advanced dementia as the review question is ‘How do informal caregivers describe their experience of providing home-based care for people with moderate to advanced dementia? Many tools have been suggested on how reviews such as this may be conducted. This review seeks to explore a broader account of informal caregivers’ experiences of dementia home-based caregiving. As such, the question coverage needs to be extensive within this scope of interest [35]. The SPIDER (Sample, Phenomenon of Interest, Design, Experience, Research type) approach, a qualitative tool for evidence synthesis will therefore be used in question definition and systematic literature interrogation. The selection of this tool is based on its specificity in investigating a defined sample’s experience of a phenomenon [32], and the possibility of achieving results of a more heterogeneous nature [11].

### Inclusion and exclusion criteria

#### Domain

Studies will be included if they are related to experiences of informal caregiving for moderate to advanced dementia.

#### Study characteristics

This review will consider all research types including evidence from qualitative, quantitative and mixed-methods studies. Only qualitative evidence from studies of qualitative or mixed-methods design is of interest, but no exclusion will be defined based on study type. All research evidence published in the English language from 1984 to present will be eligible for inclusion, given the reported increase in the diagnosis of dementia within that period. However, the possibility of achieving review objectives is dependent on the development of a clear review question and setting inclusion criteria [2, 42, 45]. Precision in framing the question therefore ensures that the population of interest is included [6], and the review question coverage is broad and extensive within the scope of interest [35]. See Table 1.

**Table 1:** Inclusion and exclusion criteria

Inclusion criteria	Exclusion criteria
Type of papers: peer-reviewed empirical papers, qualitative papers including case studies and mixed-methods (both telephone and postal surveys are acceptable only if open-ended questions are asked)	Papers that are not primary research e.g. systematic reviews, meta-analysis
Language of papers: English	Papers in other languages
Date: published between 1984 and present	Papers were written before 1984
Population of focus: unpaid family members or informal caregiver	Population of focus relates to other groups of carers e.g. paid carers, professionals
Age group: 18 and above	Below 18
Primary illness of interest: moderate to advanced dementia	Other illnesses
Setting: domestic home setting, unpaid home-based care	Other formal care establishments where care provision is paid for
Focus of papers: current experiences or views or needs of family caregiver	Focus on the views of others, or where death has already occurred

#### Sample

Informal caregivers are described as the family members, friends, relatives or anyone who provide significant care for an ill person [51]. These may be people related through committed heterosexual or same-sex partnerships, birth or adoption and others who have strong emotional and social bonds with the person receiving care. Only those acting as caregivers in an unpaid capacity will be considered. Other carers who may, or may not be family members, will be defined as lay, unpaid people in a close supportive role, who share in the illness experience of the person receiving care. Literature on other carers who do not fall within this broad definition will be excluded.

#### Phenomenon of interest

The context of interest is specific to dementia caregiving at the moderate to advanced stage within a domestic home setting. As such, it is necessary to distinguish between the home setting as a place where the person with dementia lives primarily, and other establishments where care provision may also be given, such as care homes. For the review, the informal caregiver may or may not be co-resident with the person with dementia within the home setting. Therefore, only literature on informal caregivers caring for people with a moderate to advanced dementia within this defined setting will be reviewed.

#### Design

Qualitative evidence collected from interviews, observations and surveys from studies adopting either a qualitative or mixed-methods design.

#### Experience

Subjective experiences such as views, opinions, attitudes and informal caregivers’ reasons for continued caregiving within the home setting for people with a moderate to advanced dementia will be explored. Accounts of the duration of time spent caregiving and caregivers’ feelings or emotions during care provision will also be of interest. Examples of these may include the duration of time spent providing care, relationship-based factors, isolation and feelings of guilt experienced. Only those experiences relevant to caring for people with moderate to advanced dementia will be reviewed and other experiences aimed at the general population or other illnesses will be excluded.

#### Research type

Research adopting either a qualitative or mixed-methods design which is described in terms of exposure to specific elements of caregiving responsibilities leading to a noticeable change in behaviour or wellbeing of the sample [44]. For example, this may include an indication of the duration of the exposure to which the informal caregivers were subjected, which may or may not cause a change to their health status.

#### Data sources

An initial scoping search of potentially relevant literature will be conducted to determine the distribution of relevant studies from available sources. Additional search will be conducted to identify literature in the following databases: MEDLINE Complete, CINAHL, EMBASE, PsycINFO, Web of Science and Academic Search Complete from 1984 to present. Reference lists of included papers will also be manually searched for additional relevant citations. Further searches will also be conducted on the Internet for grey literature, dissertations and theses.

### Search strategy and process

A search will be conducted by the author. The search terms and strategy will be checked by two reviewers (CW and KF), who are familiar with the domain of dementia and care provision. The search strategy will be tailored for use with each database, using Boolean operators, truncations and Medical Subject Heading (MESH) as appropriate for each database. The search strategy will use a broad range of terms and relevant keywords related to dementia, caregivers and qualitative studies. A draft search strategy for MEDLINE is included in Additional file 2.

### Data management

An electronic log will be kept of databases searched and terms used. Numerical search results will be reported using a PRISMA [33] flow diagram. EndNote bibliographic management software will be used in managing all searches including citations, abstracts and full texts. All identified papers will be directly imported through this software and duplicates can be easily identified and removed.

### Study selection process

The criteria for paper selection will follow the stipulated inclusion and exclusion criteria. Only the author will be involved in the initial paper selection. All citations will be imported to Endnote and duplicates removed before the screening. Identified studies will be screened by the two reviewers, CW and KF to ascertain that the stated inclusion criteria are met. Reviewers will not be blinded to the author’s detail, and further assistance will be sought from the librarian at Lancaster University if required. Titles and abstracts will be screened in the first stage of the selection process, to exclude ineligible studies and remove duplications. A flow chart showing details of studies included and excluded at each stage of the study selection process will be provided [33].

### Data extraction

Guidance from [40] will be followed in data extraction as well as in appraising the quality and robustness of the synthesis. In line with the search process, the decision on which data to extract would be guided by the review question, as the definition of these elements differentiates between interpretations across disparate settings and populations [40]. Key data such as author’s name, publication origin and year, setting, population and sample size, aim and objectives, illness stage, research design, data collection method and main findings, which clearly show the caregivers’ experiences would be extracted from each paper selected by the review author (see Additional file 3). A narrative summary of the synthesis will also be given as a critical reflection on the findings of each study. This would include the methodological approach followed in the synthesis, as well as the quality of data used. Any assumptions made would also be declared to ensure the credibility of the review findings.

### Quality and bias appraisal

Selected studies will be appraised by the author using the Hawker et al. [21] tool to evaluate the risk of selection bias. It is considered that the conclusions reached in reviews may be influenced by the results of individual studies [40]. It is suggested that grounds for appraising the quality of individual studies considered for review may, therefore, be defined by individual reviewers. The Hawker et al. [21] tool is selected based on its non-discriminatory capability in assessing the quality of included papers, as well as the adaptability of its structure to a variety of methodologically distinctive designs. It uses a checklist of 9 categories for assessing the quality of selected papers. The first 7 categories evaluate the trustworthiness of the paper being assessed, while the last 2 represent individual paper’s relevance. The score allocated to each study represents its weight and demonstrates the relevance, validity and appropriateness of each paper in comparison to others also selected.

On the Hawker et al. [21] tool, a maximum of 36 points is possible as a range for categorising and determining papers’ quality according to their methodological rigour. Each paper will be assigned a rating of ‘good’ (4 points), ‘fair’ (3 points), ‘poor’ (2 points) or ‘very poor’ (1 point) in the nine categories. It is considered that allocating the ratings as ‘A’, ‘B’, ‘C’, and ‘D’ instead will bring more clarity. As suggested by [29], a high-quality paper may be rated ‘A’ and scored between 30 and 36 points, a medium-quality paper may be rated ‘B’ and scored between 24 and 29, and a low-quality paper may be rated ‘C’ and scored between 9 and 24. To reduce ambiguity in this review, however, grading for a low-quality paper will be modified to a range between 9 and 23. It is not envisaged that this adaption will impact on the gradings as originally guided by [21] (see Additional file 4). The two reviewers CW and KF will check the result of the appraisal process to ensure the appropriateness of this tool.

### Data synthesis

This review adopts a narrative synthesis approach following the guidance by Popay et al. [40] for all study types identified. The use of Nvivo software will be enlisted to manage the synthesis of the data into themes. Narrative description of patterns derived from the findings from reviewed literature will be used in understanding what influences the descriptions attributed to the experiences of informal caregivers. This will involve grouping of studies and making commentary comparisons between them, using their characteristics such as their data collection methods, authors, aims and their reported findings. For clarity, the steps to undertaking the narrative synthesis by [40], suggests that the synthesis of literature be conducted by (a) identifying the review focus, (b) specifying the review question, (c) identifying studies to include (d) data extraction and quality appraisal (e) synthesising of the findings and (f) reporting of findings.

## Discussion

The focus of this review is to explore informal caregivers’ experiences of home-based caregiving for people with a moderate to advanced dementia. Psychological distress has been cited as one of their experiences and this has been associated with a negative impact on their general health. It is thought that their accounts may also suggest the experience of moral distress, which is a feeling generally associated with the powerlessness to act in situations where one knows the right thing to do. Subjective experiences such as the informal caregivers’ views, opinions, attitudes, duration of time spent caregiving and the associated reasons for continued caregiving within the home setting will be explored. Only experiences relevant to caring for people with moderate to advanced dementia will be reviewed and other experiences aimed at the general population or other illnesses will be excluded. By examining any existing knowledge within the wider literature, gaps within the wider context of caregiving in the domestic home setting will be highlighted. This may also aid further understanding and enable a proposal of interventions or support which lead to better health outcomes and quality of life for informal caregivers within the home setting.

### Potential strengths and limitations

This review considers that variation of accounts contributes to how the caregivers’ experience is explored in future research. As such, although gaps in current knowledge may be highlighted within the wider context of caregiving in the domestic home setting, full justification of accounts may be difficult as experiences will be extrapolated from a wider context within the reviewed literature. It is further considered that a limitation of the review will be its restriction to English-only sources, due to the time and resources available to the reviewers. As a qualitative endeavour, the review will however provide a rich understanding of accounts of informal caregiving within the domestic home setting. Furthermore, given that the narrow focus of the review, generalisation of findings at either of the moderate or the advanced stages may also be difficult. Transferability of those accounts across clearly defined settings and contexts is however appropriate.

## Supplementary Information

**Additional file 1:.** PRISMA-P checklist

**Additional file 2:.** MEDLINE draft search

**Additional file 3:.** Draft data extraction table

**Additional file 4:.** Quality and bias appraisal

## Data Availability

No datasets were generated or analysed during this development of this protocol. Data sharing is therefore not applicable to this article.

## Abbreviations

SPIDER: Sample, Phenomenon of Interest, Design, Experience, Research type
PRISMA-P: Preferred Reporting Items for Systematic Reviews and Meta-Analysis Protocols
NVIVO: Qualitative Analysis Software Package by QSR International
MEDLINE: Electronic database of medical journal citations and abstracts for global biomedical literature
MeSH: Medical Subject Headings
CINAHL: Cumulative Index of Nursing and Allied Health Literature
PROSPERO: International Prospective Register of Systematic Review
