# Supplementary material for: Protocol for a systematic review on the experience of informal caregivers for people with a moderate to advanced dementia within a domestic home setting
Source: Syst Rev. 2020 Nov 26;9:270. doi: 10.1186/s13643-020-01525-0 (PMC7694266; doi:10.1186/s13643-020-01525-0)
Supplement: Supplementary file 2 — Additional file 2:. MEDLINE draft search [file 13643_2020_1525_MOESM2_ESM.docx]

**Electronic search strategy conducted in MEDLINE (EBSCOhost Platform) from January 1984 to October 2020**

| **Searches** |
| --- |
| 1. MH "dementia+" |
| 2. TX dement* |
| 3. TX alzheimer* |
| 4. TX "lew* bod*" |
| 5. TX FTLD OR FTD OR frontotemporal OR vascular dement* OR mixed dement* |
| 6. 1 OR 2 OR 3 OR 4 OR 5 |
| 7. TX carer* |
| 8. TX caregiv* |
| 9. TX care-giver |
| 10. TX spouse-caregiver* |
| 11. 7 OR 8 OR 9 OR 10 |
| 12. MH "qualitative research+" |
| 13. MH qualitative research or qualitative study or qualitative methods or interview |
| 14. MH "focus groups" |
| 15. TX exploratory research |
| 16. TX ethnography qualitative research |
| 17. TX content analysis in qualitative research |
| 18. MH "grounded theory |
| 19. 12 OR 13 OR 14 OR 15 OR 16 OR 17 OR 18 |
| 20. 6 AND 11 AND 19 |

(MH = MESH Heading; TX =All Text Fields)
