# Supplementary material for: Protocol for a systematic review on the experience of informal caregivers for people with a moderate to advanced dementia within a domestic home setting
Source: Syst Rev. 2020 Nov 26;9:270. doi: 10.1186/s13643-020-01525-0 (PMC7694266; doi:10.1186/s13643-020-01525-0)
Supplement: Supplementary file 3 — Additional file 3:. Draft data extraction table [file 13643_2020_1525_MOESM3_ESM.docx]

**Data extraction table**

| Field | Description |
| --- | --- |
| Author, date, country | - First author’s name, date of publication and country of origin |
| Setting | - The setting of care provision |
| Population/sample size/sampling method/illness stage | - Caregiver’s distinctive characteristics of  - Sampling method  - Stage of illness |
| Aim/  Objectives | - Stated aims and objectives of the study |
| Research type/ research design | - Research design type |
| Data collection & analysis | - Data collection method employed |
| Findings | - Study findings showing caregiver’s experiences |
