# Supplementary material for: Protocol for a systematic review on the experience of informal caregivers for people with a moderate to advanced dementia within a domestic home setting
Source: Syst Rev. 2020 Nov 26;9:270. doi: 10.1186/s13643-020-01525-0 (PMC7694266; doi:10.1186/s13643-020-01525-0)
Supplement: Supplementary file 4 — Additional file 4:. Quality and bias appraisal [file 13643_2020_1525_MOESM4_ESM.docx]

| Field | Description |
| --- | --- |
| Author | Author’s name |
| Abstract/title | Evidence of a clear description of the study  **High quality:** Clear title and information are provided in a structured manner  **Medium quality:** Most of the information is provided  **Poor quality:** Inadequate abstract is provided |
| Introduction/  aims | Evidence of a good background to the of study and the declaration of research aims  **High quality:** Concise background and up-to date literature review is provided  **Medium quality:** The background provided is not comprehensive enough  **Poor quality:** Inadequate background is provided, and aims/objectives are also inadequate |
| Data collection | Clarity of the data collection method used is provided  **High quality:** A clear description of the method used is provided and appropriate for the purpose of the study  **Medium quality:** Method used is appropriate but inadequate description is provided  **Poor quality:** Method used is either inappropriate or there is no clear description provided |
| Sampling | Evidence of the usage of an appropriate sampling method to achieve study aims  **High quality:** Information on the sampling method includes descriptive details of participants and how they were recruited  **Medium quality:** Most details are provided but some are missing  **Poor quality:** Sampling method is mentioned but descriptive information is inadequate |
| Analysis | Clear evidence of rigor in the data analysis method  **High quality:** Good description of how analysis was conducted  **Medium quality:** Description of analysis method is inadequate  **Poor quality:** Minimal detail is provided on data analysis |
| Ethics/bias | Clear evidence of ethical approval for study conduct  **High quality:** Issue of ethics is fully addressed, and necessary approval is in place  **Medium quality:** Inadequate details provided about ethical issues  **Poor quality:** Issues of ethics were not mentioned |
| Results/findings | A clear statement of findings is provided  **High quality:** Findings are comprehensive and well presented  **Medium quality:** Inadequate details of findings are provided  **Poor quality:** Findings are not presented or poorly explained |
| Generalisability | Findings are transferable to a wider population  **High quality:** Evidence of good sampling, context and setting, allowing for the presentation of findings to be comparable to other contexts  **Medium quality:** Inadequate details are provided to allow for generalizability  **Poor quality:** Description of context and setting is poor |
| Implication | Importance of findings to policy and practice is discussed  **High quality:** Suggestion are made for policy and practice  **Medium quality:** Inadequate details provided about implications for policy and practice  **Poor quality:** Implication for policy and practice is not discussed |
| Total score | *A maximum of 36 points is possible as a range for categorising and determining papers’ quality according to their methodological rigour* |
| Grade | *Each paper will be allocated a grade of either ‘A’, ‘B’, ‘C’, or ‘D’* |

**Quality and bias appraisal**

(Source: Adapted from Hawker et al., 2002. doi: 10.1177/1049732302238251)
